# Bodies of desire: use of nonprescribed hormones among transgender women and travestis in five Brazilian capitals (2019–2021)

**DOI:** 10.1590/1980-549720240010.supl.1

**Published:** 2024-08-19

**Authors:** Katia Cristina Bassichetto, Thiago Félix Pinheiro, Claudia Barros, Paula Andrea Morelli Fonseca, Rita Suely Bacuri de Queiroz, Sandro Sperandei, Maria Amélia de Sousa Mascena Veras

**Affiliations:** I Santa Casa de São Paulo, School of Medical Sciences – São Paulo (SP), Brazil.; II Secretaria de Estado da Saúde de São Paulo, Butantan Institute – São Paulo (SP), Brazil.; III Fiocruz Amazônia,. Leônidas and Maria Deane Institute – Manaus (AM), Brasil.; IV Western Sydney University, Translational Health Research Institute – Sydney (NSW), Australia.

**Keywords:** Transgender women, Hormone, Public policies, Multicenter study

## Abstract

**Objective:**

To analyze the experiences of transgender women and *travestis* regarding the use of hormones for body changes without a medical prescription.

**Methods:**

This is a cross-sectional, quantitative and qualitative study, using data from “TransOdara”, which estimated the prevalence of Sexually Transmitted Infections in transgender women and *travestis* recruited through Respondent-Driven Sampling, between December 2019 and July 2021, in São Paulo, Campo Grande, Manaus, Porto Alegre, and Salvador, Brazil. The main outcome was: use of hormones without medical prescription and associated risk factors. Descriptive analysis, mixed univariate logistic regression models, and semi-structured interviews were carried out.

**Results:**

Of the 1,317 recruited participants, 85.9% had already used hormones. The current use of hormones was reported by 40.7% (536) of them. Of those who were able to inform the place where they obtained them, 72.6% (381/525) used them without a medical prescription. The variables associated with the outcome were: current full-time sex work (OR 4.59; 95%CI 1.90–11.06) or in the past (OR 1.92; 95%CI 1.10–3.34), not having changed their name (OR 3.59; 95%CI 2.23–5.76), not currently studying (OR 1.83; 95%CI 1.07–3.13), being younger (OR 2.16; 95%CI 1.31–3.56), and having suffered discrimination at some point in life for being a transgender women and *travestis* (OR 0.40; 95%CI 0.20–0.81).

**Conclusion:**

The use of nonprescribed hormones is high among transgender women and *travestis*, especially among those who are younger, did not study, have not changed their name, and with a history of sex work. This use is related to the urgency for gender transition, with excessive use and damage to health.

## INTRODUCTION

The transgender population is defined as that whose gender identity does not correspond to the biological sex assigned at birth^1^. This population claims the legitimacy of its diverse “non-cisgender” identities beyond the binary parameters “masculine and feminine”^2-4^. Specifically, with regard to transgender women and *travestis*, in this study we decided to address these two subgroups together. All of them constitute performances of resistance to the gender CIS(y)tem^5^.

Some transgender women and *travestis* resort to a set of gender affirmation procedures, including hormone therapy, with the aim of obtaining a body that represents the belonging to an experienced or lived gender identity^6-8^. In Brazil, to meet this demand, the Transsexualization Process was established in the Brazilian Unified Health System (SUS), through Ordinance No. 457/2008^9^, which comprised a set of care strategies focused on transgenitalization surgeries. This was regulated and expanded by Ordinance No. 2.803/2013^10^, including other medical procedures and hormone therapy in specialized care, regardless of surgery. This achievement took place through the mobilization and articulation of the social movement, in order to pressure the State to incorporate this demand^11^. However, although regulated since 2013, hormone therapy is still not universally accessible in the SUS^12^.

Studies indicate that the transgender population faces several barriers that limit the access to healthcare services, such as disrespect for their social name and difficulty dialoguing with professionals, representing important aspects of the experienced discrimination^13,14^. Such barriers produce illness, including the use of nonprescribed hormones, which is a reality in the country among the transgender women and *travestis* ^4,11,15,16^. Considering this scenario, we deemed important to analyze the experiences of transgender women regarding the use of hormones without a medical prescription for body changes and to investigate associated risk factors among participants in a multicenter study, carried out in five Brazilian capitals.

## METHODS

### Study design

The present study is an excerpt from the TransOdara Project, a cross-sectional project with a mixed approach (quantitative and qualitative), carried out between December 2019 and July 2021 in the municipalities of São Paulo, Campo Grande, Manaus, Porto Alegre, and Salvador, Brazil. Its aim was to estimate the prevalence of eight different Sexually Transmitted Infections (STIs) and understand the meanings attributed to syphilis infection among transgender women and *travestis* ^17^.

The Respondent-Driven Sampling (RDS) technique was used, which is indicated to recruit populations that are difficult to access, marginalized and sparse in terms of their insertion in the geography of urban areas^18^, despite the limitations inherent in statistical inference from non-probabilistic samples^19^, considering that the assumptions of classic probability sampling are inapplicable to studies with this population^20^.

The eligibility criteria were: male sex assigned at birth; female gender self-identification, including *travesti*, woman, transgender woman, agender, or other female gender identification; being a resident of the metropolitan areas of the participating cities, being 18 years old or over, and having received a valid study coupon from one of the previously selected “seeds.” Recruitment chains were screened using a “coupon manager.” Each participant received a unique number that identified the research location, individual identification number, and recruitment sequence.

Data were collected by trained researchers, in the locations designated for the development of the research. Of the 1,345 potential participants identified, 1,317 transgender women and *travestis* who met the eligibility criteria and signed the informed consent form were included. Subsequently, the baseline questionnaire and acceptability forms for potential prophylaxis and treatments were applied, laboratory tests were offered to detect the STIs of interest, and medical appointments were offered, with due referrals, when indicated to the prevention and treatment resources available in the SUS, on the same day of participation in the research or through later appointments.

The recruitment strategies, eligibility criteria, sample calculation, and research flow were detailed in the TransOdara methodological article in this supplement^17^.

#### Hormone use and selected variables

Only complete and consistent records were used in all analyses. The main outcome was the dichotomization (yes/no) of the use of hormones related to their transition without a medical prescription, considering only those who reported using them at the time of the interview.

“Use with a medical prescription” was considered to be those who responded affirmatively *with a prescription from a SUS doctor or a private doctor*, and “use without a medical prescription” was considered to be those who indicated *directly at the drug store (without a prescription), with friends/coworkers, “bombadeiras”* ^1^, *purchase online.*

The following were considered potential risk factors associated with the use of hormones: age group (cutoff at 35 years old); city where the research was carried out; name change on any document (yes, no); race/skin color (White, Black, mixed-race, others); currently studying (yes, no); level of education (college degree and graduate education and some graduate education; high school and some high school plus some college; and elementary school and some elementary school); monthly income (minimum wage in 2020: less than 1; 1 to 2; and 2 or more); having suffered discrimination or violence for being transgender (yes, no); self-reported mental health (very good/good, fair, poor/very poor); and history of sex work (never, only in the past; currently, part-time; currently, as main source).

### Data analysis

#### Quantitative analysis

A descriptive analysis was performed, presenting the absolute and relative frequencies for the independent variables in relation to the outcome and in general. The distribution of the use of nonprescribed hormones was described in relation to the city in which the study was carried out. Mixed univariate logistic regression models, with random intercepts for the cities, were created for each independent variable^21^, allowing adjustments for the prevalence of each city. The univariate models were used to preselect the candidate-variables used in the construction of the final multiple model, including those with p≤0.3. The selection of variables for the final multiple model considered all candidate-variables in the complete model, removed one by one, until obtaining the final model with the best adjustment according to the Akaike Information Criterion (AIC). According to Sperandei et al.^22^, the RDS weights were not used, as they do not present advantages in regression models and can add bias to the results.

The analyses were carried out in R language, version 4.2.1^23^, using the “lme4” package^24^.

#### Qualitative analysis

The narratives produced in semi-structured interviews were used, which were conducted in parallel with the quantitative component, to understand aspects that characterize the relationship of this population with meanings, practices, and healthcare services that result in greater vulnerability to syphilis and other STIs. The interview script contained questions about health conditions, gender transition, experiences with STIs, therapeutic itineraries, and the relation to healthcare services and professionals.

The majority had already participated in the quantitative stage, and contact was made during the activities carried out in this context. The selection sought to ensure diversification by categories of age, education, professional activity (i.e., sex workers *versus* other occupations) and current or previous diagnosis of syphilis. Participants were contacted via telephone and/or messages via apps, based on data provided during recruitment or other project activities. The interviews were recorded and transcribed, varying between 18 and 120 minutes.

The interpretative-comprehensive treatment of the narratives involved integrated reading with decoding of information related to the use of hormones (with and without medical follow-up), including the perspective of those who have never used such hormones. The material was analyzed together with quantitative data seeking complementarity (consonances or divergences), for deepening and expanding the articulated understanding of the results. Some excerpts are reproduced in the results section.

#### Ethical aspects

The project was approved by the Research Ethics Committee (CEP) of Santa Casa de Misericórdia de São Paulo (CAAE 05585518.7.0000.5479, No. 3.126.815 – 01/30/2019) and by the other participating institutions^17^.

## RESULTS

### Quantitative

The majority (85.9%) of the 1,317 participants in the TransOdara study reported having used hormones related to their transition at some point in their lives, with the average age at onset being 18.5 years (SD [Standard Deviation] 5.7 years). The current use of hormones was reported by 536 (40.7%) of them, of which 525 (97.9%) reported where they obtained these medications. We verified that 381 (72.6%) were using nonprescribed hormones, with wide variation between capitals (52.9% in São Paulo and 94.7% in Manaus), as shown in Table 1. The average age observed among those who reported using nonprescribed hormones was 30.7 years (SD 9.1).

**Table 1:** Distribution of the total number of transgender women and travestis, according to the use of hormones with and without medical prescription, participating in the TransOdara study, in five Brazilian capitals (December 2019 to July 2021).

Variable	With medical prescription		Without medical prescription		Total
	n	%	n	%	
São Paulo	89	47.1	100	52.9	189
Porto Alegre	30	39.0	47	61.0	77
Salvador	13	11.8	97	88.2	110
Manaus	5	5.3	89	94.7	94
Campo Grande	7	12.7	48	87.3	55
Total	144	27.4	381	72.6	525

In Table 2 we present the absolute and relative frequencies, crude odds ratio, and 95% confidence interval (CI) for the use of hormones with and without a medical prescription, according to socioeconomic and demographic characteristics of transgender women and *travestis* participating in the TransOdara. Considering only those who reported using hormones without a medical prescription at the time of the interview, most (78.3%) aged between 18 and 34 years, 72.6% had not changed their names, 67.7% reported to be Black or mixed-race, 76.5% were not studying at the time of the interview, and 72.2% had at least started high school. The majority (74.2%) had a history of sex work, with 32.0% of them only in the past and 23.5% in the present, carrying out this activity full-time. About half (44.4%) had a per capita income of up to 1 minimum wage.

**Table 2:** Absolute and relative frequencies, crude odds ratio and 95% confidence intervals for the use of hormones with and without medical prescription, according to socioeconomic and demographic characteristics of transgender women and travestis participating in the TransOdara study, in five Brazilian capitals (December 2019 to July 2021).

Variable	No	Yes	Not applicable	Total	OR	95%CI	p-value
	n (%)	n (%)	n (%)	n (%)			
Age group (in years)							
35 or over	63 (42.9)	84 (21.7)	335 (43.1)	482 (36.7)	1	- - -	- - -
Up to 34	84 (57.1)	303 (78.3)	443 (56.9)	830 (63.3)	2.38	1.53–3.72	0
Name change							
Yes	101 (68.7)	106 (27.4)	176 (22.6)	383 (29.2)	1	- - -	- - -
No	45 (30.6)	281 (72.6)	601 (77.2)	927 (70.7)	4.7	3.03–7.31	0
Race/Skin color							
White	49 (33.3)	108 (27.9)	177 (22.8)	334 (25.5)	1	- - -	- - -
Black/Mixed-race	90 (61.2)	262 (67.7)	570 (73.3)	922 (70.3)	1.02	0.64–1.62	0.933
Other	7 (4.8)	14 (3.6)	23 (3.0)	44 (3.4)	0.63	0.21–1.90	0.409
Are you currently studying?							
Yes	42 (28.6)	90 (23.3)	148 (19.0)	280 (21.3)	1	- - -	- - -
No	104 (70.7)	296 (76.5)	628 (80.7)	1028 (78.4)	1.78	1.10–2.89	0.02
Level of education							
College degree or over	16 (10.9)	18 (4.7)	31 (4.0)	65 (5.0)	1	- - -	- - -
High school (complete or some)	106 (72.1)	281 (72.6)	529 (68.0)	916 (69.8)	2.79	1.24–6.25	0.013
Elementary school (complete or some)	25 (17.0)	86 (22.2)	216 (27.8)	327 (24.9)	3.26	1.31–8.06	0.011
Sex work							
No	67 (45.6)	100 (25.8)	175 (22.5)	342 (26.1)	1	- - -	- - -
Only in the past	45 (30.6)	124 (32.0)	247 (31.7)	416 (31.7)	2.22	1.33–3.72	0.002
Current (part-time)	27 (18.4)	70 (18.1)	173 (22.2)	270 (20.6)	1.63	0.90–2.95	0.105
Current (full-time)	8 (5.4)	91 (23.5)	180 (23.1)	279 (21.3)	7.82	3.44–17.77	0
Income (in minimum wages)							
3 or over	10 (6.8)	24 (6.2)	45 (5.8)	79 (6.0)	1	- - -	- - -
2 to 3	7 (4.8)	27 (7.0)	65 (8.4)	99 (7.5)	1.63	0.49–5.41	0.424
1 to 2	56 (38.1)	110 (28.4)	260 (33.4)	426 (32.5)	1.05	0.44–2.53	0.913
<1	66 (44.9)	193 (49.9)	315 (40.5)	574 (43.8)	1.32	0.56–3.14	0.523
Discrimination							
No	15 (10.2)	70 (18.1)	103 (13.2)	188 (14.3)	1	- – -	- – -
Yes	131 (89.1)	314 (81.1)	672 (86.4)	1117 (85.1)	0.50	0.27–0.94	0.031
Verbal aggression							
No	72 (49.0)	192 (49.6)	415 (53.3)	679 (51.8)	1	- - -	- - -
Yes	75 (51.0)	192 (49.6)	353 (45.4)	620 (47.3)	0.99	0.65–1.49	0.951
Physical aggression							
No	126 (85.7)	323 (83.5)	647 (83.2)	1096 (83.5)	1	- - -	- - -
Yes	21 (14.3)	60 (15.5)	126 (16.2)	207 (15.8)	1.06	0.59–1.90	0.838
Sexual assault							
No	68 (46.3)	175 (45.2)	397 (51.0)	640 (48.8)	1	- - -	- - -
Yes	78 (53.1)	209 (54.0)	377 (48.5)	664 (50.6)	1.04	0.69–1.58	0.844
Violence							
No	8 (5.4)	41 (10.6)	58 (7.5)	107 (8.2)	1	- - -	- - -
Yes	139 (94.6)	344 (88.9)	717 (92.2)	1200 (91.5)	0.44	0.19–1.01	0.053
Mental health							
Very good	18 (12.2)	53 (13.7)	111 (14.3)	182 (13.9)	1	- - -	- - -
Good	55 (37.4)	132 (34.1)	286 (36.8)	473 (36.1)	0.99	0.51–1.95	0.986
Fair	56 (38.1)	150 (38.8)	270 (34.7)	476 (36.3)	0.97	0.50–1.90	0.933
Poor	9 (6.1)	35 (9.0)	65 (8.4)	109 (8.3)	1.45	0.54–3.88	0.458
Very poor	8 (5.4)	14 (3.6)	27 (3.5)	49 (3.7)	0.68	0.22–2.06	0.493

OR values considered statistically significant were indicated in bold.

Most of the interviewees reported having suffered discrimination (86.4%) and having experienced an episode of violence at some point in their lives because they were transgender women and *travestis* (88.9%), while 54.0% reported having suffered sexual violence. Self-perception of mental health was considered fair by 38.8% of them and poor/very poor by 12.6%. The variables positively associated with the outcome, in the bivariate analysis, were: age between 18 and 34 years; not having changed their name; not studying; whether or not they have completed Elementary or High School; and history of sex work, with greater magnitude among those who carried out this activity full-time. Having suffered discrimination at some point in life was negatively associated with the use of nonprescribed hormones.

**Figure 1:**
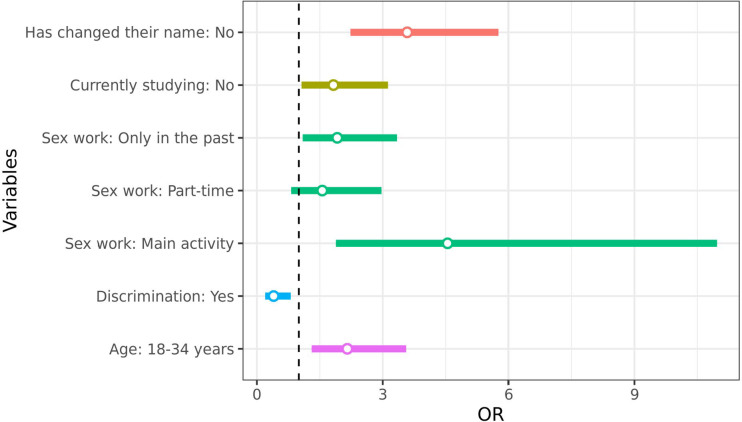
Adjusted odds ratios for the use of hormones without a medical prescription, according to socioeconomic and demographic characteristics of transgender women and travestis, participants in the TransOdara study in five Brazilian capitals (December 2019 to July 2021).

In the multiple model (Figure 1), the following variables remained associated with the outcome, with higher risks estimated at approximately 5 and 2 times, respectively: *having full-time sex work as the main occupation* (OR 4.59; 95%CI 1.90–11 .06) or *part-time* (OR 1.56; 95%CI 0.82–2.97), *in the past* (OR 1.92; 95%CI 1.10–3.34); *not having changed their name* (OR 3.59; 95%CI 2.23–5.76); *not currently studying* (OR 1.83; 95%CI 1.07–3.13); and *being younger* (18 to 34 years old), these have approximately double the risk (OR 2.16; 95%CI 1.31–3.56). Conversely, *having suffered discrimination at some point in life for being travesti*/transgender (OR 0.40; 95%CI 0.20–0.81) was negatively associated with the outcome.

### Qualitative

A total of 58 in-depth interviews were conducted, with participants between 19 and 58 years old. Some of the excerpts were selected, considering their meanings and seeking dialogue with the quantitative findings (Chart 1).

**Chart 1:** Excerpts selected from the interviewees’ narratives, with reference to fictitious names and cities (TransOdara, 2019–2021).

1. *“I’m better, I’m happier with myself when I look in the mirror, when I relate to people too. I thought* [the gender transition] *only added positive things to my life. So, I don’t regret anything, I’m going to continue with my hormone therapy, with my treatments, everything as expected. I think now I’ve found a path and I’m following it.”* (Priscila, Manaus)
2. *“In my mind, I feel like a woman inside. But on the outside, I feel the need to show […] something, but not to society. I want to show it to myself.”* (M, Salvador)
3. *“Since I was ten years old, I started taking hormones.* […] *It was with a friend of mine, whom I went to school with. So, as she was more experienced in sex, as she was no longer a virgin, she secretly took contraceptive medication, so she gave it to me and I started taking it. I started taking it and, as my body began to form, a girl’s body was formed: small breasts, hips.”* (Arlete, São Paulo)
4. *“I’ve always taken it based on the recommendation of another transgender woman, without the medical issue. The medical issue of hormones is much more recent. I’ve always taken very massive doses.”* (Andreia, Salvador)
5. *“So, today I’m 40 years old, but I started my transition very young, from 12 to 13 years old. So, back then, it was a very different thing than it is today. We didn’t have any follow-up, from any doctor. First ‘cause the doctors didn’t even understand what a transition was.”* (Michelly, São Paulo)
6. *“A friend of mine said there was this service in República* [neighborhood]*. I was already looking for something that would be more right for me to do with my body.”* (Amelita, São Paulo)
7. *“I started on my own, then taking Perlutan at the drug store. And, after a year, I sought medical advice. At the time I had Hapvida* [health insurance] *and I saw an endocrinologist from the health insurance system and there she started doing some tests, asked for some tests, and I did them. She started prescribing hormones there for me to take, estradiol, which is Sandrena gel, I kept taking it and, after a while, I lost the health insurance, so I came here, to the SUS, for follow-up. I kept being followed up, all the time doing tests and monitoring hormone levels in my body, that kind of thing. But I started on my own 4 years ago, I went through 3 years of follow-up with doctors, but the first year I did it on my own.”* (Priscilla, Manaus)
8. *“No, I’ve never taken hormones before on my own. I’ve always been very scared, because I’m very frightened of these things. They said that if we took the wrong medications, it would cause thrombosis,* […] *something would happen to the heart, it would block the vein and whatever. So, I’ve always been very afraid. And as I didn’t have much access to transgender people, I started trying to have closer transgender friends who had these friendships so I could be more educated, have more background, more awareness. And I only started taking hormones when the endocrine department at* [Hospital de] Clínicas (Clinics Hospital) *provided it to me, which are the ones I still take to date.”* (Fernanda, Porto Alegre)
9. *“The dysphoria issue is such a big deal for every* travesti*, so I said that: I’m going there* [São Paulo] *precisely to assemble myself.* […] *But I wanted it faster, I wanted to speed it up. I was already taking hormones.* […] *Society, myself, I pressured myself to conform to the heteronormative standard, that is: the more passing a transgender woman is, and the more respected she is, the less prejudice she suffers.* […] *When I started my initiation, I took 4 doses of Perlutan a month, I took two 21-day contraceptive pills a day, and I still had an Evra patch on me. Look how I was overburdening my liver and kidney.* […] *Now I’m taking a break, ‘cause in the past I tried to use Evra on my own, my God, I almost had a heart attack, and I was used to using it. Then I had a problem with an anxiety attack and it was a whole thing.”* (Michelly, São Paulo)
10. *“*[I use hormones] *on my own. I tried to be followed up, but I couldn’t.* […] *The psychologist was unable to assist me. And I ended up giving up.”* (Elis, Campo Grande)
11. *“I went there* [to the T outpatient clinic] *twice, but then it kind of stopped too, so it seems. So, I didn’t go anymore. I went there twice. And I liked it, you know? I liked it, they were going to refer me to hormones, to all sorts of things. And I want to keep going there*.” (Vitória, Porto Alegre)
12. *“I’m still at the beginning, I’ve been taking hormones for less than a month.* […] *I don’t* [do medical follow-up] . *I even wanted to start with a follow-up, but I heard that the outpatient clinic is closed, and a friend of mine, who is a hormone freak, said: ‘no biggie, every doctor will tell you to start with 1 milligram. Start taking it now, when you finish your pack, you’ll go see the doctor,’ but the outpatient clinic isn’t working. I have to go because I’m scared. I’m afraid of liver issues, ‘cause we know it can happen when it’s used inappropriately. But, for now, I’m taking it correctly, so I can do the tests and the follow up, to know whether to increase the dose or maintain it.”* (Patrícia, Campo Grande)
13. *“I use it on my own. I have a referral* [to do follow-up] *to CEDAP* [State Center for Diagnosis, Assistance and Research]*, but I haven’t been there yet to the follow up with the endocrinologist. I use it on my own. Before, I used to take injectable* [hormones]*, but I found out that with injectable* [hormones]*, the health risks are greater, so today I take pills, one a day.”* (Felipa, Salvador)

The report of the use of hormones as one of the most used resources for body changes was significant, in order to enable the desired gender affirmation and the reduction of suffering with gender dysphoria (Excerpts 1 and 2 – Chart 1). Few interviewees had never used hormones. They dispensed with this resource to avoid problems such as increased body size and weight, compromised erections, and consequent losses in sexual work.

Some participants (aged 37 years or over) historically situate the use of hormones for gender transition as a community practice developed at times when their provision and monitoring by a medical professional were not available in healthcare services, especially in the public network. According to the interviewees, this practice, generally initiated when they were very young, is originally based on knowledge disseminated among transgender women and *travestis* through sociability networks, which stand out as fundamental in the dissemination of information about the types of hormones, regimens of use, and mode of administration (Excerpts 3 to 5 – Chart 1).

The availability of hormones in healthcare services with follow-up by a medical professional appears in the narratives as a recent phenomenon, presenting itself as a possibility of using them in a safer way (Excerpts 6 to 8 – Chart 1).

Despite the availability of follow-up with a medical professional for hormone therapy, this is not always an option. In some narratives, the option for unmonitored and exaggerated use appears linked to a certain urgency in relation to gender transition, with consequent damage to health (Excerpt 9 – Chart 1). Furthermore, some barriers are referred to as hindering access to the assisted use of hormones, converging with other difficulties that make the relationship between transgender women and *travestis* and healthcare services fragile (Excerpts 10 to 12 – Chart 1).

Simultaneously, the choice to use nonprescribed hormones, even when follow-up is available, seems related to the lack of bond with healthcare services or the fear of being discriminated against in these spaces. This choice, in turn, does not necessarily imply careless or negligent use (Excerpt 13 – Chart 1).

## DISCUSSION

In this study we identified that there is a high rate of use of nonprescribed hormones for body transition of transgender women and *travestis*, especially among younger women, who were not studying, with a history of sex work, and who had not changed their name. Having suffered discrimination at some point in life for being *travesti*/transgender women was negatively associated with the outcome. The highest proportions of this behavior were observed in Manaus, Salvador, and Campo Grande.

The proportion of use of hormones at some point in life, in the present study (85.9%), was lower than that observed in San Francisco – USA (94.0%)^25^ and in Salvador – state of Bahia, Brazil (94.8%)^4^ and higher than the *Transcendendo* cohort study in Rio de Janeiro – RJ (45.7%)^26^. In relation to the current use of hormones, the proportion observed in this study (40.7%) was similar to that found in Canada (43.0%)^27^ and lower than that found in San Francisco – USA (49.1%)^25^, in Rio de Janeiro (49.1%)^26^ and Salvador (69.0%)^4^, Brazil.

Regarding the prevalence of current use of nonprescribed hormones (72.6%), the main outcome of the present study, we verified that, in comparison with national studies, it was lower than that of *Divas* – Federal District (84.0%)^28^, similar to that of *Transcendendo* – Rio de Janeiro (78.7%)^26^, to that of *Pop Trans* – Salvador (69.1%)^4^, to that of *Muriel* – seven municipalities in the state of São Paulo (79.4%)^16^, and higher than that of transgender women and *travestis* of *Trans* – Universidade do Estado do Rio de Janeiro – UERJ (53.6%)^15^. Considering international studies, the prevalence was lower than that found in Lima – Peru (88.4%)^29^ and higher than that verified in Boston – USA (4.2%)^30^, Ontario – Canada (26.8%)^27^, and San Francisco – USA (49.1%)^25^.

Several factors may explain the differences found in the prevalence of hormone self-medication. Authors of some international studies focused on the transgender population already linked to healthcare services^27,30^, while most national studies recruited transgender women through RDS^4,16,26,28^. Other possible factors can be highlighted, such as: temporality of the study; study locations; different legislation on the implementation of these services and access barriers, such as the capacity of municipalities to expand the provision of services; unpreparedness of professionals; experienced stigma; lack of inputs in the field of public health; low socioeconomic status; and belief that a quantity of hormones greater than prescribed would cause faster body changes^3,4,25,28^. The meanings attributed to the use of hormones are relevant in gender affirmation processes, with the potential to reduce suffering and increase the well-being of transgender women and *travestis*, especially with regard to self-perception, self-esteem, and social interactions^31^. These findings are in line with the statement of Nascimento^5^, according to whom “bodies are references that can function as an anchor for our identities, a firm point to which we bond and connect” [free translation]. Therefore, the process of gender affirmation involves addressing several subjectivities, with the use of hormones being an important tool for building the desired body^31^.

Several factors have been positively associated with the use of nonprescribed hormones, such as: having undergone reassignment surgery and having experienced verbal aggression due to gender identity and expression^26^; negative experiences with healthcare professionals; limited financial resources; lack of healthcare services related to gender transition and having undergone surgery with own financial resources^27^; and lack of inputs in the field of public health and unpreparedness of professionals regarding the prescription of these medications for transgender women^32^. In three national studies^4,25-27,31,32^ and one international^26^, the factors that showed the greatest association with the use of nonprescribed hormones were similar: being younger and history of sex work.

The interviewees’ narratives indicate that the high rate of use of nonprescribed hormones reflects barriers to accessing services, the desire for a quick transition, and the shortage of trained professionals. Although the literature on the topic is limited, these findings are similar to those of the systematic review carried out by Kennedy et al.^33^

The TransOdara findings point to low coverage of healthcare services that meet specific needs and a lack of preparation of healthcare professionals. Based on the participants’ reports, we can infer that the relatively recent implementation of gender transition procedures in the SUS is still insufficient. Conversely, self-medication seems to be consolidated in the cultural scene of transgender people, with an important characteristic being the centrality of women’s networks in the dissemination of information and in the administration of nonprescribed hormones, as also observed in Lima — Peru^29^ and in Bogotá — Colombia^34^.

The dissemination of information among transgender women and *travestis* and peer support in accessing services emerge in the participants’ reports, indicating the relevance of networks for expanding the assisted use of these medications, improving bonds with such services, and reducing risks and complications resulting from the use of hormones without adequate prescription. Authors of a recent literature review found adverse effects of using excessive doses of these medications among transgender women such as circulation problems and mood changes^35^. These women, who purchase hormones without a prescription, have little information about dosage, efficacy, contraindications, and adverse effects^29^.

### Study limitations

The first limitation of the study is inherent in the cross-sectional design, whose primary objective was to investigate the prevalence of selected STIs, limiting the in-depth analysis of issues such as hormone therapy. The second limitation refers to the method used to recruit participants (RDS), considering that, despite being widely used in studies with populations that would be difficult to recruit using other sampling methods, the obtained results must be interpreted with caution, as they can be only representative of the sociability networks eventually captured by the study, whose characteristics, in general, are unknown. In the absence of better strategies, RDS continues to be a method of choice for studies with populations of transgender women in various contexts^36^.

Another limitation consisted of the difficulties of conducting the research during the new coronavirus (COVID-19) pandemic, resulting in interruptions in recruitment chains, changing locations for carrying out the research, and difficulties establishing references to immediately meet the participants’ demands.

### Final considerations

Despite the aforementioned difficulties, this is a study with robust numbers, which showed the high use of nonprescribed hormones in transgender women and *travestis* from five capitals representing the Brazilian macro-regions. The findings reinforce results from previous studies^4,16^, whose authors already highlighted this serious public health issue. The persistence of important barriers of access to the use of prescribed hormones does not comply with the expected role of the State so that this population can benefit from this right.

Reducing the prevalence of the use of nonprescribed hormones is urgent, requiring several actions, from structural initiatives — to avoid stigmatizing and discriminatory attitudes — to a thorough analysis of the implementation of those in force, to identify gaps and flaws to be corrected, especially among younger women, with a history of sex work, who do not study, who have not yet changed their names, and who suffer discrimination for being transgender. The set of factors associated with the use of nonprescribed hormones in the TransOdara study evidences individual, social, and structural vulnerabilities that negatively impact the health of this population in several dimensions.

In order to improve care for this population, continuous training of multidisciplinary teams is expected at all SUS levels to provide adequate guidance on the correct use of hormones and follow up treatment, aiming at reducing adverse events^35^; the implementation and expansion of the number of specialized services in all Brazilian regions; broad communication of the rights guaranteed by law, especially the use of social names, providing respectful and nondiscriminatory service, among others.

Beyond the scope of this analysis, understanding the consequences of the use of hormones for the health of transgender people remains a current and relevant issue, which should be deepened in other studies.

## Footnotes

1
*Travestis* or transgender women, usually older, who master the technique for applying industrial silicone (known as “bombar”/“bombação” in Portuguese) in other *travestis*/transgender women with the aim of changing their bodies.
