# Supplementary material for: Bodies of desire: use of nonprescribed hormones among transgender women and travestis in five Brazilian capitals (2019–2021)
Source: Rev Bras Epidemiol. 2024 Aug 19;27(Suppl 1):e240010.supl.1. doi: 10.1590/1980-549720240010.supl.1 (PMC11338539; doi:10.1590/1980-549720240010.supl.1)
Supplement: Translation

# Translation: Corpos do desejo: uso de hormônios sem prescrição médica entre mulheres trans e travestis em cinco capitais brasileiras (2019–2021)

**DOI:** 10.1590/1980-549720240010.supl.1.2

**Authors:** Katia Cristina Bassichetto, Thiago Félix Pinheiro, Claudia Barros, Paula Andrea Morelli Fonseca, Rita Suely Bacuri de Queiroz, Sandro Sperandei, Maria Amélia de Sousa Mascena Veras

**Affiliations:** I Santa Casa de São Paulo, Faculdade de Ciências Médicas – São Paulo (SP), Brasil.; II Secretaria de Estado da Saúde de São Paulo, Instituto Butantan – São Paulo (SP), Brasil.; III Fiocruz Amazônia,. Instituto Leônidas e Maria Deane – Manaus (AM), Brasil.; IV Western Sydney University, Translational Health Research Institute – Sydney (NSW), Australia.

**Keywords:** Mulher transexual, Hormônios, Políticas em saúde pública, Estudo multicêntrico

## Abstract

**Objective:**

Analisar experiências de mulheres trans e travestis com o uso de hormônios para mudança corporal sem prescrição médica.

**Métodos:**

Estudo de corte transversal, quantitativo e qualitativo, utilizando um recorte do TransOdara, que estimou prevalência de Infecções Sexualmente Transmissíveis em mulheres trans e travestis, recrutadas por meio de *Respondent-Driven Sampling*, entre dezembro de 2019 e julho de 2021, em São Paulo, Campo Grande, Manaus, Porto Alegre e Salvador. Principal desfecho: uso de hormônios sem prescrição médica e fatores associados. Foram realizados análise descritiva e modelos de regressão logísticos univariados mistos e entrevistas semiestruturadas.

**Resultados:**

Das 1.317 participantes recrutadas, 85,9% já haviam usado hormônios. O uso atual de hormônios foi referido por 40,7% (536) delas. Das que souberam informar o local onde os conseguiram, 72,6% (381/525) faziam uso sem prescrição médica. As variáveis associadas ao uso sem prescrição foram: exercer trabalho sexual em tempo integral (OR 4,59; IC95% 1,90–11,06) ou no passado (OR 1,92; IC95% 1,10–3,34); não ter retificado o nome (OR 3,59; IC95% 2,23–5,76); não estar estudando (OR 1,83; IC95% 1,07–3,13); ser mais jovem (OR 2,16; IC95% 1,31–3,56); ter sofrido discriminação em algum momento na vida por ser mulheres trans e travestis (OR 0,40 – IC95% 0,20–0,81).

**Conclusão:**

O uso de hormônios não prescritos é alto entre mulheres trans e travestis, principalmente entre as mais jovens, as que não estudavam, as que não retificaram o nome e as com histórico de trabalho sexual. Está relacionado à urgência para a transição de gênero, com uso exagerado e danos à saúde.

## INTRODUÇÃO

População trans é definida como aquela cuja identidade de gênero não corresponde ao sexo biológico atribuído ao nascimento^1^. Essa população reivindica a legitimidade de suas diversas identidades “não cisgêneras” para além dos parâmetros binários “masculino e feminino”^2-4^. Especificamente, no que se refere às mulheres trans e travestis, optou-se, neste estudo, por tratar esses dois subgrupos conjuntamente. Todas se constituem como performances de resistência ao CIStema de gênero^5^.

Parte das mulheres trans e travestis recorrem a um conjunto de procedimentos de afirmação de gênero, entre eles a hormonização, com a finalidade de obter um corpo que represente o pertencimento a uma identidade de gênero experenciada ou vivida^9^, que compreendia um conjunto de estratégias assistenciais, focado nas cirurgias de transgenitalização. Este foi regulamentado e ampliado pela Portaria n^10^, incluindo outros procedimentos médicos e hormonioterapia na atenção especializada, independentemente da cirurgia. Essa conquista se deu pela mobilização e articulação do movimento social, a fim de pressionar o Estado a incorporar essa demanda^11^. Entretanto, embora regulamentada desde 2013, a hormonização ainda não é acessível de forma universal no SUS^12^.

Estudos apontam que a população trans enfrenta diversas barreiras que limitam o acesso aos serviços de saúde, como o desrespeito ao nome social e a dificuldade de diálogo com os profissionais, representando importantes aspectos da discriminação vivenciada^13,14^. Tais barreiras produzem adoecimento, incluindo o uso de hormônios não prescritos, que é uma realidade no país entre a população trans^4,11,15,16^. Diante desse cenário, considerou-se importante analisar as experiências das mulheres trans e travestis sobre uso de hormônios sem prescrição médica para mudança corporal e investigar fatores de risco associados entre participantes de um estudo multicêntrico, realizado em cinco capitais brasileiras.

## MÉTODOS

### Desenho

O presente estudo é um recorte do Projeto TransOdara, transversal com abordagem mista (quantitativa e qualitativa), realizado entre dezembro de 2019 e julho de 2021 em São Paulo, Campo Grande, Manaus, Porto Alegre e Salvador. Teve como objetivo estimar a prevalência de oito diferentes Infecções Sexualmente Transmissíveis (ISTs) e compreender os significados atribuídos à infecção por sífilis entre mulheres trans e travestis^17^.

Foi utilizada a técnica de amostragem *Respondent-Driven Sampling* (RDS), indicada para recrutar populações de difícil acesso, marginalizadas e esparsas, em termos de sua inserção na geografia das áreas urbanas^18^, a despeito das limitações inerentes à inferência estatística de amostras não probabilísticas^19^, dado que os pressupostos da amostragem probabilística clássica são inaplicáveis a estudos com essa população^20^.

Os critérios de elegibilidade foram: sexo masculino atribuído ao nascer; autoidentificação de gênero feminina, incluindo travesti, mulher, mulher trans, agênero ou outra identificação do gênero feminino; ser residente nas áreas metropolitanas das cidades participantes ter idade igual ou superior a 18 anos e ter recebido um cupom do estudo válido de uma das “sementes” selecionadas previamente. As cadeias de recrutamento foram rastreadas usando um “gerenciador de cupons”. Cada participante recebeu um número exclusivo que identificava o local da pesquisa, o número de identificação individual e a sequência de recrutamento.

A coleta de dados foi realizada por pesquisadores(as) treinados(as), nos locais designados para o desenvolvimento da pesquisa. Das 1.345 potenciais participantes identificadas, foram incluídas 1.317 mulheres trans e travestis que atenderam aos critérios de elegibilidade e assinaram o termo de consentimento livre e esclarecido. Na sequência, foram aplicados o questionário *baseline* e os formulários de aceitabilidade para potenciais profilaxias e tratamentos, oferecidos testes laboratoriais para detecção das ISTs de interesse e consultas médicas, com os devidos encaminhamentos, quando indicados para recursos de prevenção e tratamento disponíveis no SUS, no mesmo dia da participação na pesquisa ou por meio de agendamentos posteriores.

As estratégias de recrutamento, critérios de elegibilidade, cálculo da amostra e fluxo da pesquisa foram detalhados no artigo metodológico do TransOdara neste suplemento^17^.

#### Uso de hormônio e variáveis selecionadas

Somente registros completos e consistentes foram utilizados em todas as análises. O principal desfecho foi a dicotomização (sim/não) do uso de hormônios relacionados à sua transição sem prescrição médica, considerando apenas quem referiu estar em uso no momento da entrevista.

Considerou-se como “uso com prescrição médica” as que responderam afirmativamente *com receita de médico do SUS ou de médico particular* e “uso sem prescrição médica” as que indicaram *diretamente na farmácia (sem receita), com amigos/colegas de trabalho, com bombadeiras, compra pela internet.*

Foram considerados potenciais fatores de risco, associados ao uso de hormônios: faixa etária (corte em 35 anos); cidade de realização da pesquisa; retificação do nome em algum documento (sim, não); raça/cor de pele (branca, preta, parda, outras); estudando atualmente (sim, não); escolaridade (ensino superior completo e pós-graduação completa e incompleta, ensino médio completo e incompleto mais superior incompleto e ensino fundamental completo e incompleto); renda mensal (salário-mínimo em 2020: menos de 1, 1 a 2 e 2 ou mais); ter sofrido discriminação ou violência por ser trans (sim, não); saúde mental autorreferida (muito boa/boa, moderada, ruim/muito ruim) e histórico de trabalho sexual (nunca, apenas no passado; atual, de modo parcial; atual, como fonte principal).

### Análise dos dados

#### Análise quantitativa

A análise descritiva foi realizada, apresentando as frequências absolutas e relativas para as variáveis independentes em relação ao desfecho e de forma geral. A distribuição do uso de hormônios não prescritos foi descrita em relação à cidade de realização do estudo. Modelos de regressão logísticos univariados mistos, com interceptos aleatórios para as cidades, foram criados para cada variável independente^21^, permitindo ajustes para as prevalências de cada cidade. Os modelos univariados foram utilizados para pré-seleção das variáveis-candidatas utilizadas na construção do modelo múltiplo final, incluindo-se aquelas com p≤0.3. A seleção das variáveis para o modelo múltiplo final considerou todas as variáveis-candidatas no modelo completo, retiradas uma a uma, até obtenção do modelo final com melhor ajuste pelo Akaike Information Criterion (AIC). De acordo com Sperandei et al.^22^, os pesos RDS não foram utilizados, uma vez que não apresentam vantagens em modelos de regressão e podem adicionar vieses aos resultados.

As análises foram realizadas em linguagem R, versão 4.2.1^23^, utilizando o pacote “lme4”^24^.

#### Análise qualitativa

Utilizou-se as narrativas produzidas em entrevistas semiestruturadas, conduzidas paralelamente ao componente quantitativo, para compreender aspectos que caracterizam a relação dessa população com significados, práticas e serviços de saúde que resultam numa maior vulnerabilidade à sífilis e a outras ISTs. O roteiro de entrevista continha questões sobre condições de saúde, transição de gênero, experiências com ISTs, itinerários terapêuticos e relação com serviços e com profissionais de saúde.

A maioria já havia participado da etapa quantitativa, e o contato foi feito durante as atividades realizadas nesse âmbito. A seleção buscou assegurar uma diversificação por categorias de idade, escolaridade, atuação profissional (i.e., profissionais do sexo *versus* outras ocupações) e diagnóstico atual ou prévio de sífilis. As participantes foram contatadas por telefone e/ou mensagens via aplicativos, a partir dos dados fornecidos no recrutamento ou em outras atividades do projeto. As entrevistas foram gravadas e transcritas, variando entre 18 e 120 minutos.

O tratamento interpretativo-compreensivo das narrativas envolveu leitura integrada com decodificação de informações relativas ao uso de hormônios (com e sem acompanhamento médico), incluindo a perspectiva daquelas que nunca fizeram tal uso. O material foi analisado em articulação com os dados quantitativos na busca de complementaridade (consonâncias ou divergências), de aprofundamento e ampliação na compreensão articulada dos resultados. Alguns excertos são reproduzidos na seção de resultados.

#### Aspectos éticos

O projeto foi aprovado pelo Comitê de Ética em Pesquisa (CEP) da Santa Casa de Misericórdia de São Paulo (CAAE 05585518.7.0000.5479, n° 3.126.815 – 30/01/2019) e pelas demais instituições participantes^17^.

## RESULTADOS

### Quantitativos

A maioria (85,9%) das 1.317 participantes do estudo TransOdara relatou já ter feito uso de hormônios relacionados à sua transição em algum momento da vida, sendo a idade média de início 18,5 anos (DP 5,7 anos). O uso atual de hormônios foi referido por 536 (40,7%), dentre as quais 525 (97,9%) informaram o local onde conseguiram esses medicamentos. Constatou-se que 381 (72,6%) faziam uso de hormônios não prescritos, com ampla variação entre as capitais (52,9% em São Paulo e 94,7% em Manaus), conforme apresentado na Tabela 1. A média de idade observada entre aquelas que afirmaram fazer uso de hormônios não prescritos foi de 30,7 anos (DP 9,1).

**Tabela 1:** Distribuição do total de mulheres trans e travestis, segundo uso de hormônios com e sem prescrição médica, participantes do estudo TransOdara em cinco capitais brasileiras (dezembro de 2019 a julho de 2021).

Variável	Com prescrição médica		Sem prescrição médica		Total
	n	%	n	%	
São Paulo	89	47,1	100	52,9	189
Porto Alegre	30	39,0	47	61,0	77
Salvador	13	11,8	97	88,2	110
Manaus	5	5,3	89	94,7	94
Campo Grande	7	12,7	48	87,3	55
Total	144	27,4	381	72,6	525

Na Tabela 2 são apresentadas as frequências absolutas e relativas, *odds ratio* bruto e IC95% do uso de hormônios com e sem prescrição médica, segundo características socioeconômicas e demográficas de mulheres trans e travestis participantes do TransOdara. Considerando apenas as que referiram estar em uso de hormônio sem prescrição médica no momento da entrevista, a maioria (78,3%) tinha entre 18 e 34 anos, 72,6% não tinham retificado o nome, 67,7% se autodefiniram como pretas ou pardas, 76,5% não estavam estudando no momento da entrevista e 72,2% tinham pelo menos iniciado o Ensino Médio. A maioria (74,2%) tinha histórico de trabalho sexual, sendo 32,0% delas apenas no passado e 23,5% no presente, exercendo essa atividade em período integral. Cerca da metade (44,4%) tinha renda *per capita* de até 1 salário-mínimo.

**Tabela 2:** Frequências absolutas e relativas, *odds ratio* bruto e IC95% do uso de hormônios com e sem prescrição médica, segundo características socioeconômicas e demográficas de mulheres trans e travestis participantes do estudo TransOdara, em cinco capitais brasileiras (dezembro de 2019 a julho de 2021).

Variável	Não	Sim	Não se aplica	Total	OR	IC95%	Valor de p
	n (%)	n (%)	n (%)	n (%)			
Faixa etária (em anos)							
35 ou mais	63 (42,9)	84 (21,7)	335 (43,1)	482 (36,7)	1	- - -	- - -
Até 34	84 (57,1)	303 (78,3)	443 (56,9)	830 (63,3)	2,38	1,53–3,72	0
Retificação do nome							
Sim	101 (68,7)	106 (27,4)	176 (22,6)	383 (29,2)	1	- - -	- - -
Não	45 (30,6)	281 (72,6)	601 (77,2)	927 (70,7)	4,7	3,03–7,31	0
Raça/cor							
Branca	49 (33,3)	108 (27,9)	177 (22,8)	334 (25,5)	1	- - -	- - -
Preta/parda	90 (61,2)	262 (67,7)	570 (73,3)	922 (70,3)	1,02	0,64–1,62	0,933
Outra	7 (4,8)	14 (3,6)	23 (3,0)	44 (3,4)	0,63	0,21–1,90	0,409
Estuda atualmente?							
Sim	42 (28,6)	90 (23,3)	148 (19,0)	280 (21,3)	1	- - -	- - -
Não	104 (70,7)	296 (76,5)	628 (80,7)	1028 (78,4)	1,78	1,10–2,89	0,02
Escolaridade							
Ensino superior ou mais	16 (10,9)	18 (4,7)	31 (4,0)	65 (5,0)	1	- - -	- - -
Ensino médio (completo ou incompleto)	106 (72,1)	281 (72,6)	529 (68,0)	916 (69,8)	2,79	1,24–6,25	0,013
Ensino fundamental (completo ou incompleto)	25 (17,0)	86 (22,2)	216 (27,8)	327 (24,9)	3,26	1,31–8,06	0,011
Trabalho sexual							
Não	67 (45,6)	100 (25,8)	175 (22,5)	342 (26,1)	1	- - -	- - -
Somente no passado	45 (30,6)	124 (32,0)	247 (31,7)	416 (31,7)	2,22	1,33–3,72	0,002
Atual (parcial)	27 (18,4)	70 (18,1)	173 (22,2)	270 (20,6)	1,63	0,90–2,95	0,105
Atual (tempo integral)	8 (5,4)	91 (23,5)	180 (23,1)	279 (21,3)	7,82	3,44–17,77	0
Renda (em salários mínimos)							
3 ou mais	10 (6,8)	24 (6,2)	45 (5,8)	79 (6,0)	1	- - -	- - -
2 a 3	7 (4,8)	27 (7,0)	65 (8,4)	99 (7,5)	1,63	0,49–5,41	0,424
1 a 2	56 (38,1)	110 (28,4)	260 (33,4)	426 (32,5)	1,05	0,44–2,53	0,913
<1	66 (44,9)	193 (49,9)	315 (40,5)	574 (43,8)	1,32	0,56–3,14	0,523
Discriminação							
Não	15 (10,2)	70 (18,1)	103 (13,2)	188 (14,3)	1	- – -	- – -
Sim	131 (89,1)	314 (81,1)	672 (86,4)	1117 (85,1)	0,50	0,27–0,94	0,031
Agressão verbal							
Não	72 (49,0)	192 (49,6)	415 (53,3)	679 (51,8)	1	- - -	- - -
Sim	75 (51,0)	192 (49,6)	353 (45,4)	620 (47,3)	0,99	0,65–1,49	0,951
Agressão física							
Não	126 (85,7)	323 (83,5)	647 (83,2)	1096 (83,5)	1	- - -	- - -
Sim	21 (14,3)	60 (15,5)	126 (16,2)	207 (15,8)	1,06	0,59–1,90	0,838
Agressão sexual							
Não	68 (46,3)	175 (45,2)	397 (51,0)	640 (48,8)	1	- - -	- - -
Sim	78 (53,1)	209 (54,0)	377 (48,5)	664 (50,6)	1,04	0,69–1,58	0,844
Violência							
Não	8 (5,4)	41 (10,6)	58 (7,5)	107 (8,2)	1	- - -	- - -
Sim	139 (94,6)	344 (88,9)	717 (92,2)	1200 (91,5)	0,44	0,19–1,01	0,053
Saúde mental							
Muito boa	18 (12,2)	53 (13,7)	111 (14,3)	182 (13,9)	1	- - -	- - -
Boa	55 (37,4)	132 (34,1)	286 (36,8)	473 (36,1)	0,99	0,51–1,95	0,986
Regular	56 (38,1)	150 (38,8)	270 (34,7)	476 (36,3)	0,97	0,50–1,90	0,933
Ruim	9 (6,1)	35 (9,0)	65 (8,4)	109 (8,3)	1,45	0,54–3,88	0,458
Muito ruim	8 (5,4)	14 (3,6)	27 (3,5)	49 (3,7)	0,68	0,22–2,06	0,493

Em negrito foram apontados os valores de OR considerados estatisticamente significativos.

A maior parte das entrevistadas relatou ter sofrido discriminação (86,4%) e ter tido algum episódio de violência alguma vez na vida por ser mulheres trans e travestis (88,9%), enquanto 54,0% referiram ter sofrido violência sexual. A autopercepção da saúde mental foi considerada regular por 38,8% delas e ruim/muito ruim por 12,6%. As variáveis positivamente associadas ao desfecho, na análise bivariada, foram: idade entre 18 e 34 anos; não ter retificado o nome; não estar estudando; ter ou não concluído o Ensino Fundamental ou Médio; e histórico de trabalho sexual, com maior magnitude entre aquelas que exerciam essa atividade em tempo integral. Ter sofrido discriminação na vida foi associada negativamente ao uso de hormônios sem prescrição.

No modelo múltiplo (Figura 1) se mantiveram associados ao desfecho, com maiores riscos estimados em cerca de 5 e de 2 vezes, respectivamente: *ter como principal ocupação o trabalho sexual full time* (OR 4,59; IC95% 1,90–11,06) ou *parcialmente* (OR 1,56; IC95% 0,82–2,97), *no passado* (OR 1,92; IC95% 1,10–3,34); *não ter retificado o nome* (OR 3,59; IC95% 2,23–5,76); *não estar estudando* (OR 1,83; IC95% 1,07–3,13); e serem *mais jovens (18 a 34 anos),* estas com cerca do dobro de risco (OR 2,16; IC95% 1,31–3,56). Por outro lado, *ter sofrido discriminação alguma vez na vida por ser travesti/transexual* (OR 0,40; IC95% 0,20–0,81) apresentou associação negativa com o desfecho.

**Figura 1:** *Odds ratios* ajustados de uso de hormônios sem prescrição médica, segundo características socioeconômicas e demográficas de mulheres trans e travestis, participantes do estudo TransOdara em cinco capitais brasileiras (dezembro de 2019 a julho de 2021).

### Qualitativos

Foram realizadas 58 entrevistas em profundidade, com participantes entre 19 e 58 anos. Alguns dos excertos foram selecionados, considerando os seus sentidos e significados e buscando diálogo com os achados quantitativos (Quadro 1).

**Quadro 1:** Excertos selecionados de narrativas das participantes entrevistadas, com referência aos nomes fictícios e cidades (TransOdara, 2019–2021).

1. *“Eu estou melhor, estou mais feliz comigo mesma ao me olhar no espelho, me relacionar com as pessoas também. Eu achei que* [a transição de gênero] *só veio acrescentar em coisas positivas para minha vida. Assim, eu não estou arrependida de nada, vou continuar com essa minha terapia hormonal, com meus tratamentos, tudo direitinho. Eu acho que agora eu encontrei um caminho e estou indo.”* (Priscila, Manaus)
2. *“Pela mente, eu me sinto uma mulher por dentro. Mas por fora eu sinto a necessidade de mostrar […] algo, mas não para a sociedade. Eu quero mostrar para mim.”* (M, Salvador)
3. *“Desde os dez anos, eu comecei a tomar hormônio. (…) Foi com uma amiga minha, que eu estudava. Aí, como ela era mais experiente no sexo, que ela já não era mais virgem, ela tomava remédio escondido, anticoncepcional, aí ela me deu e eu comecei a tomar. Eu comecei a tomar e, quando o meu corpo foi se formando, se formou um corpo de menina: peitinho, quadril.”* (Arlete, São Paulo)
4. *“Eu sempre tomei por indicação de outra trans, sem a questão médica. A questão médica dos hormônios é muito mais recente. Eu sempre tomei doses muito maciças”.* (Andreia, Salvador)
5. *“Então, hoje eu tenho 40 anos de idade, mas eu iniciei a minha transição muito nova, de 12 para 13 anos de idade. Então, naquela época, era uma coisa bem diferente do que é hoje. A gente não tinha acompanhamento nenhum, de médico nenhum. Primeiro que os médicos nem entendiam o que era uma transição.”* (Michelly, São Paulo)
6. *“Uma amiga minha falou que tinha esse serviço na República. Eu já estava procurando algo que seria mais certo de se fazer com meu corpo.”* (Amelita, São Paulo)
7. *“Eu comecei por conta própria, então aplicando Perlutan na farmácia. E, depois de um ano, eu procurei orientação médica. Na época eu tinha o Hapvida e fui com uma endócrino do sistema do plano de saúde e lá ela começou a fazer os exames, passou os exames, e eu fiz. Ela começou a receitar hormônio por lá para eu tomar, o estradiol, que é o Sandrena gel, eu fiquei tomando, e, depois de um tempo, eu perdi o plano, aí eu vim para cá, para o SUS, para acompanhamento. Já ficou direto sendo acompanhada, todo tempo fazendo exame e monitorando os níveis de hormônio no corpo, essa questão aí. Mas eu comecei por conta própria há 4 anos, passei por 3 anos de acompanhamento com os doutores, mas o primeiro ano foi sozinha mesmo.”* (Priscilla, Manaus)
8. *“Não, nunca tomei hormônios antes por conta. Eu sempre tive muito medo, porque eu sou muito cagona com essas coisas. Eles diziam que se a gente tomasse medicamentos errados dava trombose, (…) dava algo no coração, entupia a veia, não sei o quê. Então eu sempre tive muito medo. E como eu não tinha muito acesso a pessoas trans, eu comecei a tentar ter amigos trans mais próximos que tinham essas amizades para poder ter mais estudo, mais bagagem, mais consciência. E eu só comecei a tomar hormônio quando o endócrino do* [Hospital de] *Clínicas me forneceu, que são os que eu tomo até hoje.”* (Fernanda, Porto Alegre)
9. *“A questão da disforia de toda a travesti é uma coisa tão grande, que aí eu falei assim, eu vou para lá* [São Paulo] *justamente para me montar toda. (…) Mas eu queria aquilo mais rápido, queria acelerar. Eu estava já tomando hormônio. (…) A sociedade, eu me cobrava para entrar no padrão heteronormativo, que seria: quanto mais passável uma trans é, e mais respeitada ela é, menos preconceito ela tem. (…) Quando comecei a minha iniciação, eu tomava 4 doses de Perlutan no mês, tomava duas pílulas de ciclo 21 por dia, e ainda grudava adesivo de Evra em mim. Olha a sobrecarga que eu fazia no meu fígado e no meu rim. (…) Agora estou dando uma parada, porque no passado eu fui inventar de tomar Evra por conta própria, meu Deus, eu tive quase um princípio de infarto, e eu era acostumada a tomar. Depois eu tive problema de crise de ansiedade e foi um babado”.* (Michelly, São Paulo)
10. *“*[Uso hormônios] *por conta própria. Tentei fazer acompanhamento, não consegui. (…) O psicólogo não conseguiu me atender. E eu acabei desistindo.”* (Elis, Campo Grande)
11. *“Eu fui duas vezes lá* [ao ambulatório T]*, mas aí, meio que deu uma parada também, parece. E aí eu não fui mais. Eu fui duas vezes lá. E eu gostei, entendeu? Eu gostei, eles iam me encaminhar para hormônios, para um monte de coisa. E eu quero continuar indo lá*.” (Vitória, Porto Alegre)
12. *“Eu estou no início ainda, não faz nem 1 mês que eu estou tomando hormônio. (…) Não* [faço acompanhamento médico]*. Eu queria, inclusive, começar com acompanhamento, mas eu soube que o ambulatório está fechado, e uma amiga minha, que é a louca dos hormônios, ela falou: ‘não tem problema, todo médico vai falar para você começar com 1 miligrama. Começa a tomar agora, quando você encerrar a sua cartela, você vai atrás do médico’, só que não está funcionando o ambulatório. Eu tenho que ir porque eu tenho medo. Eu tenho medo de dar problema no fígado, porque a gente sabe que dá, quando o uso é inapropriado. Mas, por enquanto, eu estou tomando certinho, para fazer os exames e fazer o acompanhamento, para saber se aumenta a dose ou mantém.”* (Patrícia, Campo Grande)
13. *“Faço uso por conta própria. Eu tenho encaminhamento para fazer no Sedap, mas só que eu não fui lá ainda para fazer esse acompanhamento com a endocrinologista. Eu faço uso por conta própria. Antes eu tomava injetado, só que eu fiquei sabendo que os injetáveis, os riscos para a saúde são maiores, aí hoje eu tomo pílula, uma por dia.”* (Felipa, Salvador)

Foi expressivo o relato de uso de hormônios como um dos recursos mais utilizados para as mudanças corporais, de modo a possibilitar a afirmação de gênero almejada e a diminuição de sofrimento com disforia de gênero (Excertos 1 e 2 – Quadro 1). Poucas entrevistadas nunca usaram hormônios. Estas dispensaram esse recurso para evitar problemas como aumento do tamanho e peso corporais, comprometimento da ereção e consequentes prejuízos no trabalho sexual.

Algumas participantes (com 37 anos ou mais) situam historicamente o uso de hormônios para transição de gênero como uma prática comunitária desenvolvida em épocas em que a sua oferta e o acompanhamento com profissional médico não estavam disponíveis nos serviços de saúde, sobretudo na rede pública. Segundo as entrevistadas, essa prática, iniciada, em geral, quando muito jovens, é originalmente baseada num saber disseminado entre mulheres trans e travestis por meio das redes de sociabilidade, que se destacam como fundamentais na difusão de informações acerca dos tipos de hormônios, esquemas de uso e modo de administração (Excertos 3 a 5 – Quadro 1).

A disponibilidade de hormônios em serviços de saúde com acompanhamento com profissional médico aparece nas narrativas como um fenômeno recente, apresentando-se como uma possibilidade de usá-los de forma mais segura (Excertos 6 a 8 – Quadro 1).

Apesar da disponibilidade de acompanhamento com profissional médico para hormonização, este nem sempre se efetiva como uma opção. Em algumas narrativas, a opção pelo uso sem acompanhamento e exagerado aparece vinculada a certa urgência em relação à transição de gênero, com consequentes danos à saúde (Excerto 9 – Quadro 1). Além disso, algumas barreiras são referidas como dificultadoras do acesso ao uso assistido de hormônios, convergindo com outras dificuldades que tornam frágil a relação das mulheres trans e travestis com os serviços de saúde (Excertos 10 a 12 – Quadro 1).

Ao mesmo tempo, a escolha pelo uso sem prescrição, mesmo quando a oferta de acompanhamento está disponível, aparece relacionada à falta de vínculo com os serviços de saúde ou ao receio de serem discriminadas nesses espaços. Essa escolha, por sua vez, não implica necessariamente o uso despreocupado ou descuidado (Excerto 13 – Quadro 1).

## DISCUSSÃO

Este estudo identificou que é alto o indicativo de uso de hormônios não prescritos para transição corporal de mulheres trans e travestis, principalmente entre as mais jovens, que não estavam estudando, com histórico de trabalho sexual e que não haviam retificado o nome. Ter sofrido discriminação alguma vez na vida por ser travesti/mulheres trans esteve associado negativamente ao desfecho. As mais altas proporções desse comportamento foram observadas em Manaus, Salvador e Campo Grande.

A proporção de uso de hormônios em algum momento da vida, no presente estudo (85,9%), foi inferior à observada em São Francisco – EUA (94,0%)^25^ e em Salvador – BA (94,8%)^4^ e superior ao estudo de coorte Transcendendo no Rio de Janeiro – RJ (45,7%)^26^. Em relação ao uso atual de hormônios, a proporção observada neste estudo (40,7%) foi semelhante à verificada no Canadá (43,0%)^27^ e inferior às verificadas em São Francisco – EUA (49,1%)^25^, no Rio de Janeiro (49,1%)^26^ e em Salvador (69,0%)^4^.

Quanto à prevalência do uso atual de hormônio sem prescrição médica (72,6%), principal desfecho do presente estudo, de forma comparativa a estudos nacionais, verificou-se que foi inferior à do Divas – Distrito Federal (84,0%)^28^, semelhante à do Transcendendo – Rio de Janeiro (78,7%)^26^, à do Pop Trans – Salvador (69,1%)^4^, à de Muriel – sete municípios do estado de São Paulo (79,4%)^16^ e; superior à de mulheres trans e travestis do Trans – Universidade do Estado do Rio de Janeiro – UERJ (53,6%)^15^. Considerando os estudos internacionais, a prevalência foi inferior à verificada em Lima – Peru (88,4%)^29^ e superior à de Boston – EUA (4,2%)^30^, Ontario – Canadá (26,8%)^27^ e São Francisco – EUA (49,1%)^25^.

Diversos fatores podem explicar as diferenças encontradas nas prevalências de automedicação de hormônios. Alguns estudos internacionais focaram na população trans já vinculada a serviços de saúde^27,30^, enquanto a maioria dos nacionais recrutou mulheres trans por meio de RDS^4,16,26,28^. Outros possíveis fatores podem ser destacados, tais como: temporalidade do estudo; locais de realização; diferentes legislações sobre a implantação desses serviços e barreiras de acesso, como capacidade dos municípios para ampliar a oferta de serviços; despreparo dos profissionais; estigma vivenciado; falta de insumos no âmbito da saúde pública; baixa condição socioeconômica e crença de que uma quantidade de hormônios maior do que a prescrita provocaria uma mudança corporal mais rápida^3,4,25,28^. Os sentidos atribuídos ao uso de hormônios são relevantes nos processos de afirmação de gênero, com potencial de diminuição do sofrimento e aumento do bem-estar de mulheres trans e travestis, especialmente no que se refere à autopercepção, à autoestima e às interações sociais^31^. Esses achados vão ao encontro da afirmação de Nascimento^5^ de que “os corpos são referências que podem funcionar como âncora para nossas identidades, um ponto firme ao qual nos vinculamos e nos conectamos”. Dessa forma, o processo de afirmação de gênero passa pelo atendimento de diversas subjetividades, sendo o uso de hormônios uma importante ferramenta para a construção do corpo desejado^31^.

Vários fatores têm sido associados positivamente ao uso de hormônios não prescritos, como: ter feito a cirurgia de redesignação e ter tido experiência de agressão verbal em função de sua identidade e expressão de gênero^26^; experiências negativas com profissionais de saúde; recursos financeiros limitados; ausência de serviços de saúde relacionados à transição de gênero e ter feito cirurgia com recursos financeiros próprios^27^; e falta de insumos no âmbito da saúde pública e despreparo dos profissionais sobre a prescrição desses medicamentos para mulheres trans^32^. Em três estudos nacionais^4,25-27,31,32^ e em um internacional^26^, os fatores que apresentaram maior associação ao uso de hormônios não prescritos foram semelhantes: serem mais jovens e mencionarem trabalho sexual.

As narrativas das entrevistadas indicam que o alto índice de uso de hormônios sem prescrição reflete as barreiras de acesso aos serviços, o desejo de uma transição rápida e a escassez de profissionais capacitados. Ainda que limitada a literatura sobre o tema, tais achados são semelhantes aos da revisão sistemática realizada por Kennedy et al.^33^

Os achados do TransOdara apontam para baixa cobertura de serviços de saúde que atendam às necessidades específicas e falta de preparo dos profissionais de saúde. Do relato das participantes, pode-se deduzir que a relativamente recente implementação dos procedimentos de transição de gênero no SUS é ainda insuficiente. Por outro lado, a automedicação parece estar consolidada na cena cultural das pessoas trans, tendo como característica importante a centralidade das redes de mulheres na disseminação de informações e na própria administração não prescrita de hormônios, como também observado em Lima — Peru^29^ e em Bogotá — Colômbia^34^.

A divulgação de informações entre mulheres trans e travestis e o apoio de pares no acesso a serviços aparecem no relato das participantes, indicando a relevância das redes para a ampliação do uso assistido desses medicamentos, a melhora do vínculo com tais serviços e a redução de riscos e complicações decorrentes do uso sem prescrição adequada dos hormônios. Uma recente revisão verificou efeitos adversos do uso de doses exageradas desses medicamentos entre mulheres trans, como problemas de circulação e alterações de humor^35^. Essas mulheres, que adquirem hormônios sem prescrição, têm pouca informação sobre posologia, eficácia, contraindicações e efeitos adversos^29^.

### Limitações do estudo

A primeira limitação do estudo é inerente ao desenho de natureza transversal, cujo objetivo primário foi a investigação das prevalências das ISTs selecionadas, limitando o aprofundamento de questões como a hormonização. A segunda refere-se ao método utilizado para o recrutamento das participantes (RDS), dado que, apesar de ser amplamente utilizado em estudos com populações que seriam dificilmente recrutadas por outros métodos amostrais, os resultados obtidos precisam ser interpretados com cautela, pois podem ser representativos apenas das redes sociais eventualmente captadas pelo estudo, cujas características, em geral, são desconhecidas. Na ausência de melhores estratégias, o RDS continua a ser um método de eleição para estudos com populações de mulheres trans em vários contextos^36^.

Outra limitação foi as dificuldades da condução durante a pandemia de COVID-19, resultando em interrupções das cadeias de recrutamento, mudança de local para realização da pesquisa e dificuldades em estabelecer as referências para atendimento imediato às demandas das participantes.

### Considerações finais

Não obstante as dificuldades mencionadas, este é um estudo com números robustos, que revelou o alto uso de hormônios sem prescrição em mulheres trans e travestis de cinco capitais representativas das macrorregiões brasileiras. Os achados reforçam resultados de estudos anteriores^4,16^, que já apontavam esse grave problema de saúde pública. A persistência de barreiras importantes de acesso ao uso de hormônios prescritos está em dessintonia com o papel esperado do Estado para que essa população possa se beneficiar desse direito.

Reduzir a prevalência do uso de hormônios não prescritos é premente, sendo necessárias diversas ações, desde estruturais, para evitar atitudes estigmatizantes e discriminatórias, até a análise minuciosa da implementação daquelas vigentes, para identificar lacunas e falhas a serem corrigidas, especialmente entre as mais jovens, com histórico de trabalho sexual, que não estudam, que ainda não retificaram os seus nomes e que sofrem discriminação por serem trans. O conjunto de fatores associados ao uso de hormônios sem prescrição no TransOdara revela vulnerabilidades de natureza individual, social e estrutural que impactam negativamente a saúde dessa população em várias dimensões.

A fim de melhorar a assistência a essa população, é esperado, em todos os níveis do SUS, o treinamento contínuo de equipes multiprofissionais para que forneçam orientação adequada sobre o uso correto dos hormônios e monitorem o tratamento, visando, inclusive, à redução de eventos adversos^35^; a implantação e ampliação do número de serviços especializados em todas as regiões brasileiras; a ampla comunicação dos direitos assegurados em lei, especialmente o uso do nome social, proporcionando um atendimento respeitoso e sem discriminação, entre outros.

Para além do escopo desta análise, a compreensão das consequências do uso de hormônios para a saúde de pessoas trans permanece como questão atual e relevante, que merece ser aprofundada em outros estudos.
